# Local Host Adaptation and Use of a Novel Host in the Seed Beetle *Megacerus eulophus*

**DOI:** 10.1371/journal.pone.0053892

**Published:** 2013-01-11

**Authors:** Gisela C. Stotz, Lorena H. Suárez, Wilfredo L. Gonzáles, Ernesto Gianoli

**Affiliations:** 1 Departamento de Botánica, Universidad de Concepción, Concepción, Chile; 2 Departamento de Ciencias Biológicas y Fisiológicas, Universidad Peruana Cayetano Heredia, Lima, Perú; 3 Departamento de Biología, Universidad de La Serena, La Serena, Chile; University of Massachusetts, United States of America

## Abstract

Spatial variation in host plant availability may lead to specialization in host use and local host adaptation in herbivorous insects, which may involve a cost in performance on other hosts. We studied two geographically separated populations of the seed beetle *Megacerus eulophus* (Coleoptera: Bruchidae) in central Chile: a population from the host *Convolvulus chilensis* (in Aucó) and a population from *C. bonariensis* (in Algarrobo). In Aucó *C. chilensis* is the only host plant, while in Algarrobo both *C. bonariensis* and *C*. *chilensis* are available. We tested local adaptation to these native host plants and its influence on the use of another, exotic host plant. We hypothesized that local adaptation would be verified, particularly for the one-host population (Aucó), and that the Aucó population would be less able to use an alternative, high-quality host. We found evidence of local adaptation in the population from *C. chilensis*. Thus, when reared on *C. chilensis*, adults from the *C. chilensis* population were larger and lived longer than individuals from the *C. bonariensis* population, while bruchids from the two populations had the same body size and longevity when reared on *C. bonariensi*s. Overall, bruchids from the *C. chilensis* population showed greater performance traits than those from the *C. bonariensis* population. There were no differences between the bruchid populations in their ability to use the alternative, exotic host *Calystegia sepium*, as shown by body size and longevity patterns. Results suggest that differences in local adaptation might be explained by differential host availability in the study populations.

## Introduction

Plants are heterogeneous environments for herbivorous insects [1]. Spatial and temporal variation in availability and quality of resources may result in differential use of host plants, which can lead to specialization in host use. In fact, most phytophagous insects are rather specialized in terms of host range [2]–[4]. The degree of specialization often changes along the distribution range of herbivorous insects [5], [6], partly because their geographic distribution differs from that of their host plants. This results in localities where there are only few hosts available [7], [8]. Thus, insect species considered generalists may behave as specialists at a population level [5].

Specialization in host use by phytophagous insects may lead to local adaptation to the host plant; thus, local individuals exhibit greater fitness components such as performance, survival or reproduction in their home environment (plant) compared to foreign individuals [9]. Local adaptation is thought to evolve by natural selection [10], being generated when populations in different environments respond to distinct selective pressures. Such divergent selection would promote the evolution of traits that provide fitness benefits for local populations under the prevailing conditions, regardless of the fitness consequences of these phenotypes in other habitats [9]. Variation in host plant availability may drive divergent selection [11], as has been shown for the leaf beetle *Oreina elongata* [12]. For insects that are seed predators, such as bruchid beetles (Coleoptera: Bruchidae), previous studies have shown that host plant chemistry [13], seed size [14], seed hardness [15], and competition [16] can be agents of selection.

Individuals from a locally adapted population should exhibit greater use efficiency on their host plant as compared to alternative hosts [17]; conversely, a generalist genotype is expected to show a similar degree of adaptation to available host plants [9]. Because of the complexity of patterns of host use in phytophagous insects, it cannot be assumed that, for a given host, specialists will have greater efficiencies in resource use than generalists [5], [18], [19]. It is often found that insect adaptation to a particular host comes at a cost of reduced performance on other hosts [18], [19], [20], [21]. For instance, in the seed beetle *Stator limbatus*, specialization on a lower quality host decreases its performance on other hosts [7]. However, there are also studies showing that locally adapted genotypes are able to maintain, and even improve, their fitness on other hosts or environments [12], [22]–[24].

Bruchid beetles (Coleoptera: Bruchidae) represent a good model system to test hypotheses on local adaptation and contrasting performance between generalist and specialist species or populations. Bruchids oviposit on seeds and their entire development occurs inside a single seed, being thus easy to manipulate experimentally [25], [26]. Seed characteristics have been shown to influence the evolution of body size and life history traits in bruchids [7], [8], [14], [26], which often show specialization in host use [27] and local adaptation [8]. In the present study, working with the native bruchid *Megacerus eulophus*, we tested local adaptation on host-plants and its influence on the use of another, exotic host in central Chile. Bruchids from *Megacerus* genus are restricted to seeds of the Convolvulaceae plant family [28]. Specifically, the system consists of two geographically separated populations of *M. eulophus* from different host plants: *Convolvulus chilensis* (in Aucó, an interior location) and *C. bonariensis* (in Algarrobo, a coastal location). The localities have different host plant availability for these seed beetles. In Aucó *C. chilensis* is the only host plant, while in Algarrobo both *C. bonariensis* and *C*. *chilensis* are available. A previous study showed significant differences in fecundity, longevity and body size between these *M. eulophus* populations, but not in seed size or nitrogen content between the two host plant species [29]. We hypothesized that local adaptation would be verified in this system, and that it would be of greater magnitude in the one-host population (Aucó) than in the two-host population (Algarrobo). We further expected that the Aucó population would be less able to use an alternative host.

## Materials and Methods

*Megacerus eulophus* Erichson (Bruchidae) is a bruchid beetle distributed along South America. The entire *Megacerus* genus only uses seeds from the Convolvulaceae plant family (morning glory family), including plants from the genera *Argyreia*, *Calystegia*, *Convolvulus*, *Ipomoea* and *Merremia* [28], [30], [31]. In Chile, where this study was conducted, *M. eulophus* has been recorded in host plants from the genera *Convolvulus*, *Ipomoea* and *Calystegia* (Convolvulaceae) [32]. Female bruchids oviposit on the external wall of mature fruits, sepals and dispersed seeds. Egg hatching starts 4–8 days after oviposition (at 24–25 °C) and the first larval stage burrows into the seed. The larva consumes almost the whole seed content. Larval and pupal development occurs completely inside a single seed. Adults emerge after 25–30 days [29].

Beetles were obtained from seeds gathered in two localities in central Chile: Aucó (31°30’ S, 71°06’ W, 600 m a.s.l), an interior location, and Algarrobo (33°21’ S, 71°39’ W, 1 m a.s.l), a coastal location, and reared in the laboratory. The distance between locations is 214 km. All necessary permits were obtained for the described field studies. A permit to conduct fieldwork in Aucó, which is located within Las Chinchillas National Reserve, was obtained from CONAF (National Forestry Corporation; Auth. Fondecyt 1030702). A permit to carry out research in Algarrobo was granted by Algarrobo town council (Decree N°2269). In Aucó we collected seeds of *Convolvulus chilensis*, the only available host, where *M. eulophus* may infest up to 50% of the seed crop [29]. In Algarrobo we collected seeds of *C. bonariensis*, the more abundant host plant, but *C. chilensis* was also available as host. We also collected seeds of *Calystegia sepium* (*Calystegia*, hereafter) in Concepción (36°46′ S, 73°03′ W, 12 m a.s.l). A permit to do field work in this site, which is located within university property, was obtained from the Universidad de Concepción. Seeds of *C. sepium* (∼30 mg) are much larger than those of *C. chilensis* and *C. bonariensis* (∼18 mg in both cases) [14], [29]. Seeds were collected from more than 20 mother plants of each species. *C. chilensis* is a perennial herb endemic to northern-central Chile and typical of coastal and arid zones, often occurring in small populations [33], [34]. *C. bonariensis* is a perennial herb native to southern South America that is morphologically very similar to *C. chilensis* and occurs in scattered populations along the coast of central Chile ([35],Gianoli and Suárez, personal observations). In both *Convolvulus* species fruits are ovoid, glabrous capsules that contain one to four seeds [35]. *Calystegia* is a perennial plant from the northern hemisphere that in Chile is common in humid microsites in central and southern of Chile [36], [37] and is used as host plant by *M. eulophus* [32], [34].

We checked daily for bruchid emergence from seeds of *C. chilensis* and *C. bonariensis*. Virgin males and females were paired randomly within each group. Each couple was confined separately in a Petri dish (50 mm Ø) in the laboratory (24–25 °C, 12 h daylength). Couples were reared in the seeds from their respective host species of origin and the emerging insects represented the F1 generation, which was subjected to the experimental treatments.

### Local Adaptation Experiment

F1 couples of *M. eulophus* from *C. chilensis* (Aucó) were randomly assigned to two rearing treatments: (1) seeds of *C. chilensis* (original host), and (2) seeds of *C. bonariensis* (alternative host, not present in the original habitat). Likewise, F1 couples from *C. bonariensis* (Algarrobo) were assigned to (1) seeds of *C. bonariensis* (original host) and (2) seeds of *C. chilensis* (alternative host, present in the original habitat). In all treatments, five seeds were initially provided and, as the insects oviposited, clean seeds replaced those carrying eggs. Thus, four groups were obtained in the F2 generation. In the F2, we worked with 51 to 86 individuals per group, including males and females. Bruchids were fed with a honey:pollen solution (9∶1) following earlier rearing procedures [29].

When F2 individuals died we recorded longevity (total adult lifespan) and body size (pronotum area). The area of pronotum (the dorsal aspect of the first thoracic segment) was used as body size estimate, as done in earlier studies with *M. eulophus* [29]. Size measures were made on digital pictures using image analysis software (SigmaScan Pro 5.0, Systat Software Inc, Richmond, CA, USA). Beetles were photographed under 20x magnification.

### Alternative Host Experiment

*Calystegia* could be considered a high-quality host because its seeds are significantly larger than those of *C. chilensis* and *C. bonariensis* [14], [29], and greater seed mass in *Calystegia* has been associated with higher fecundity and greater offspring size in *M. eulophus* [14]. However, we have no specific data about the relative performance of *M. eulophus* in native hosts vs. *Calystegia*. For this experiment we worked with F1 individuals of *M. eulophus*, which had been grown in their respective hosts of origin: *C. chilensis* and *C. bonariensis*. Half of the bruchid couples from *C. chilensis* and *C. bonariensis* were offered with seeds of *Calystegia* to oviposit, and the other half of the couples from each population received seeds from their respective hosts, from which we obtained an F2 generation. Five seeds were initially given and, as the insects oviposited, seeds were replaced by clean ones. The F2 generation was fed with honey:pollen solution (9∶1). We obtained 55 individuals from the *C. chilensis* population and 76 individuals from the *C. bonariensis* population, both reared on *Calystegia* seeds. When these individuals died, we recorded longevity (total adult lifespan) and body size (pronotum area).

### Data Analysis

In both experiments variation in body size and longevity was analyzed using GLMs. To analyze longevity, body size was entered as a covariate. The main factors in the experiment of local adaptation were population of origin (Population) and rearing host (Environment). Longevity was log-transformed before the analysis to meet the normal distribution assumption. Local adaptation is detected in significant Population x Environment interactions for the bruchid performance variables. In the alternative host experiment the main factor was the population of origin.

## Results

**Figure 1:**
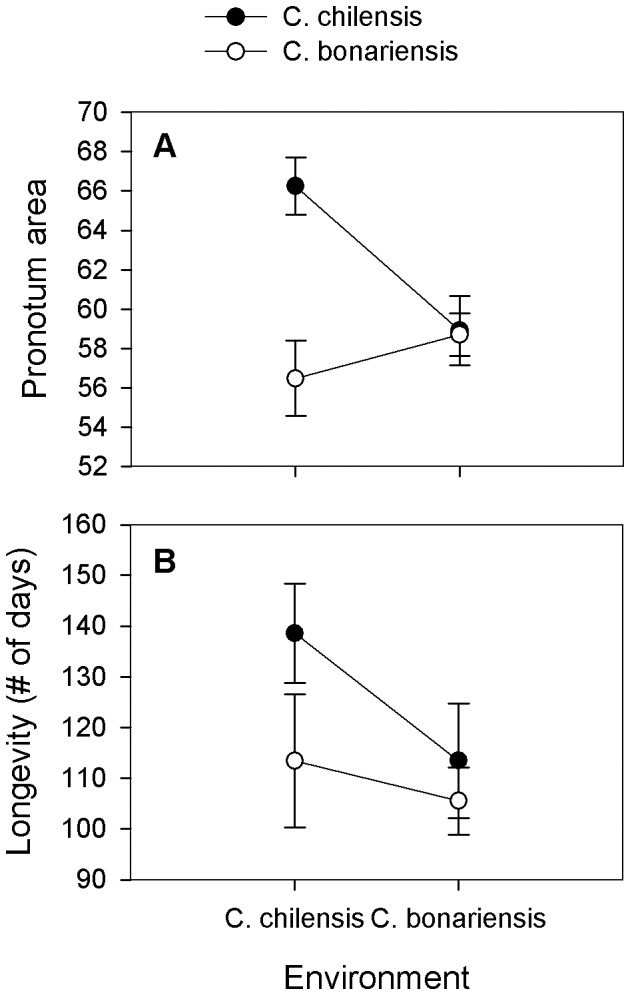
Local adaptation of two populations of *Megacerus eulophus*. Performance of the bruchid beetle *Megacerus eulophus* on the host plants *Convolvulus chilensis* and *C. bonariensis* (Environment). Bruchids were collected in field populations of *C. chilensis* (closed circles) and *C. bonariensis* (open circles) and reared in the experimental hosts for two generations. Performance traits were Body size (Pronotum area) (A) and Longevity (B). Means ± SE are shown.

We found evidence of local adaptation in the population from *C. chilensis* for both variables measured: body size and longevity (Table 1, Fig. 1). Thus, when reared on *C. chilensis*, individuals from the *C. chilensis* population were larger than individuals from the *C. bonariensis* population, while bruchids from the two populations had very similar body size when reared on *C. bonariensi*s (Fig. 1A). Likewise, when reared on *C. chilensis*, individuals from the *C. chilensis* population lived longer than those from the *C. bonariensis* population, and there was little difference in longevity between populations when bruchids were reared on *C. bonariensis* (Table 1, Fig. 1B). Overall, bruchids from the *C. chilensis* population showed greater performance traits than those from the *C. bonariensis* population (Fig. 1).

**Table 1:** Local adaptation: GLM analysis on performance traits of *Megacerus eulophus*.

	Pronotum area	Longevity
Population (P)	10.337 **	2.941 ns
Environment (E)	2.721 ns	1.091 ns
P x E	9.531 **	4.895 *

ns P>0.05;

* P<0.05;

** P<0.01;

*** P<0.001.

Chi-square values are shown (df = 1 for all variables) after a General Lineal Model (GLM) analysis. Effect of host plant of origin (Population) and host plant of rearing (Environment) on performance traits of the seed beetle *Megacerus eulophus* from the host plants *Convolvulus chilensis* and *C. bonariensis*. Pronotum area, the variable of body size, was entered as covariate in the Longevity analysis.

**Figure 2:**
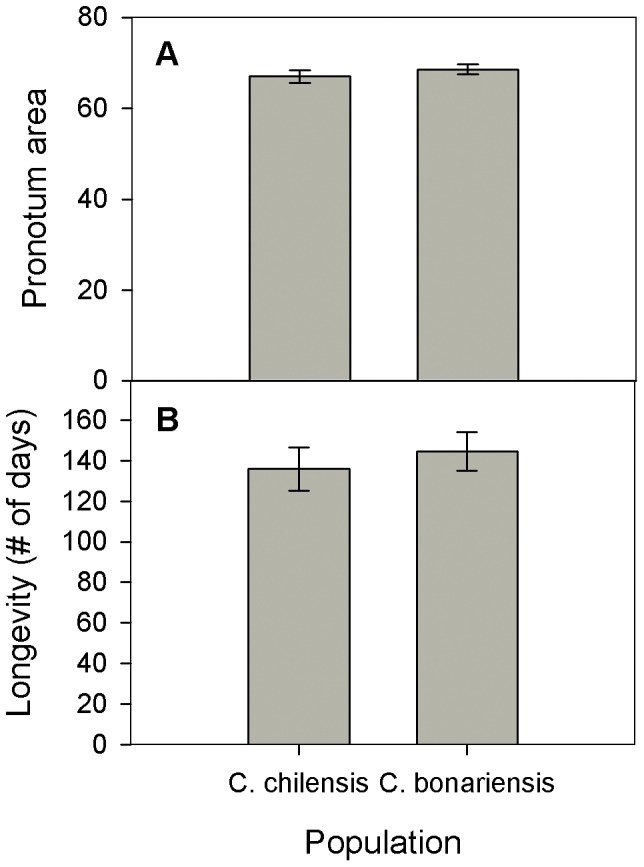
Performance of two populations of *Megacerus eulophus* in an alternative host. Performance of the bruchid beetle *Megacerus eulophus* from populations of the host plants *Convolvulus chilensis* and *C. bonariensis* when reared on a novel host, *Calystegia sepium*. Performance traits were Body size (Pronotum area) (A) and Longevity (B). Means ± SE are shown.

There were no differences between the bruchid populations in their ability to use the alternative, exotic host *Calystegia*. The *C. chilensis* and *C. bonariensis* populations of *M. eulophus* showed the same body size (Chi-square = 0.7494, p-value = 0.3867, df = 1) and longevity (Chi-square = 0.9297, p-value = 0.3349, df = 1) when reared on *Calystegia* seeds (Fig. 2). This pattern resulted from the maintenance and increase of performance traits for the *C. chilensis* and *C. bonariensis* populations, respectively (inspection of Figs. 1 and 2).

## Discussion

Following the “local vs. foreign” approach to the study of local adaptation suggested by [9], we found –as expected– evidence consistent with local adaptation only for the population of *M. eulophus* originally from *C. chilensis*. In particular, bruchids from this population had larger body size and lived longer when reared on *C. chilensis* seeds as compared to those reared on *C. bonariensis*. Differential host performance between different populations may be a first step towards reproductive isolation [38]–[41], as shown recently for the bruchid *Callosobruchus maculatus* [42]. It is therein discussed that host-preference and assortative mating can coevolve in the absence of local adaptation, but in order to maintain assortative mating after secondary contact, fitness differences and local adaptation appear to be necessary [42]. Interestingly, the two host plant species (*C. chilensis* and *C. bonariensis*) do not differ in seed size or seed nitrogen content [29], which are general indicators of host quality for seed beetles [14], [43]–[45]. Thus, it is likely that the observed pattern of local host adaptation in bruchid performance results from the specific exploitation of a particular nutritional factor [46] or to differences in seed coat properties [47]. These hypotheses deserve further investigation.

Maternal effects could be considered to play a role in the observed patterns, because local adaptation may be facilitated if female’s host experience influences offspring performance in these hosts [48]. However, inspection of results indicates that in this case maternal effects are unlikely to be involved: no consistent effect of maternal host on bruchid performance was observed. A previous study on *M. eulophus* showed that it was seed size, and not the maternal environment, what mainly affected offspring size [14], and in the present case host seed did not differ in size. Nevertheless, we cannot rule out the occurrence of maternal effects during the adaptation of *M. eulophus* to one of its host plants (*C. chilensis*), as has been reported for other bruchid beetles [49], [50].

Differential host availability in the study locations might explain –at least in part– the contrasting results regarding local adaptation. In Aucó, *C. chilensis* is the only host plant available for *M. eulophus*. Consequently, *C. chilensis* probably exerts a directional selective pressure towards adaptation to this specific host plant. In Algarrobo, where both host plants are available, a pressure for divergent selection should be weaker. Despite it is often recognized that geographic differences in host availability may be important for the occurrence of local (host) adaptation [6], [39], [41], [51], there is scant empirical evidence supporting this hypothesis. The influence of host plant availability on local host adaptation has been demonstrated in the leaf beetle *Oreina elongata*, but only for one of the traits measured (insect growth rate) [12]. We report here two traits, body size and longevity, supporting this hypothesis in the case of the seed beetle *Megacerus eulophus*. Other studies have not found evidence of host availability influencing local host adaptation in the seed beetle *Stator limbatus* [7], [8] and in the stem borer *Apagomerella versicolor* [52].

The fact of being locally adapted to their host plant seemingly did not limit bruchids from the *C. chilensis* population in the use of an alternative, exotic host (*Calystegia*). These bruchids and those from the *C. bonariensis* population showed similar performance in the new host plant. Thus, in the present study system insect adaptation to a particular host did not come at a significant cost in performance on other hosts, as it is generally hypothesized [7], 19–21,23,24. However, considering that i) for the population from *C. chilensis* none of the performance traits where we found evidence of local adaptation (body size and longevity) increased when the population was reared on the alternative host *Calystegia*, and ii) in the local adaptation experiment the performance of bruchids from the *C. chilensis* population was shown to be, overall, greater than that of their conspecifics, these results may also be interpreted otherwise. Thus, it could be suggested that the processes underlying local adaptation in the *C. chilensis* population of *M. eulophus* bruchids prevented them from taking full advantage of *Calystegia*, a putative high-quality host. In contrast insects from the *C. bonariensis* population did increase their performance when reared on *Calystegia* seeds. These results may be partially explained by the hosts’ distribution, because the distribution of *Calystegia* spans the location of the *C. bonariensis* population but not that of the *C. chilensis* population [37]. Bruchids from the C. bonariensis population had a higher chance of encountering a population of *Calystegia* in the past and this it is less likely to be a novel host plant for them. Results may not be explained by phylogenetic relatedness among host plants, because *C. chilensis* and *C. bonariensis* are closely related species, and the alternative host plant (*Calystegia*) belongs to another genus. Further experiments including other Convolvulaceae host plants used by *M. eulophus* in Chile, and of varying relatedness with *C. chilensis*, would shed light on this issue.

Bruchids from both populations were able to use *Calystegia*, although with differential success. *Calystegia* is an exotic plant that arrived to central Chile about 150 years ago [36]. Considering that *Calystegia* is much more abundant than the native *Convolvulus* species, it would be interesting to study the long term adaptation of *M. eulophus* (particularly for the bruchid from the *C. bonariensis* population) to this exotic host and another equally abundanct host, C. arvensis ( [32], [36], Gianoli, personal observation). It would be of interest to determine whether this adaptation process brings about changes in life history and/or morphological traits, as has been reported for the soapberry bug *Jadera haemolotoma* after colonizing an introduced host plant from the same family [53].

Together with host quality experienced by bruchids during the larval stages [54], [55], the maternal environment may also significantly influence bruchid life history and fitness [49], [50], [56], [57]. In particular, it has been shown for *M. eulophus* that maternal diet quality affected egg size plasticity [14]. As discussed above, it is unlikely that the results observed are consequences of carry-over effects from the maternal hosts, which did not differ in seed attributes and are closely related species. In conclusion, this study has found evidence of local host adaptation in the population of *M. eulophus* where *C. chilensis* is the only host, and no such a pattern was found for the population from *C. bonariensis*, where *C. chilensis* is also available as host. Thus, these differences might be explained by local host availability in the study populations. We further found preliminary evidence that local adaptation did not significantly prevent the use of an alternative host by the *C. chilensis* population of the bruchid *M. eulophus*. Further research should address whether host preference patterns match the performance patterns reported here.
