# Correction: Local Host Adaptation and Use of a Novel Host in the Seed Beetle *Megacerus eulophus*

**DOI:** 10.1371/annotation/fa8ff99f-9052-4173-aed3-03e482976667

**Published:** 2013-09-09

**Authors:** Gisela C. Stotz, Lorena H. Suárez, Wilfredo L. Gonzáles, Ernesto Gianoli

The fourth author was incorrectly omitted from the list of those authors who wrote the paper in the Contributions Statement. The Author Contributions section should read: “Conceived and designed the experiments: EG LHS WLG. Performed the experiments: LHS WLG. Analyzed the data: GCS. Contributed reagents/materials/analysis tools: EG. Wrote the paper: GCS EG." 

